# Physical activity and preventable premature deaths from non-communicable diseases in Brazil

**DOI:** 10.1093/pubmed/fdy183

**Published:** 2018-10-20

**Authors:** Leandro Fórnias Machado de Rezende, Leandro Martin Totaro Garcia, Grégore Iven Mielke, Dong Hoon Lee, Edward Giovannucci, José Eluf-Neto

**Affiliations:** 1 Departamento de Medicina Preventiva, Faculdade de Medicina FMUSP, Universidade de Sao Paulo, Sao Paulo, SP, Brazil; 2 UKCRC Centre for Diet and Activity Research, MRC Epidemiology Unit, University of Cambridge School of Clinical Medicine, Cambridge, UK; 3 School of Human Movement and Nutrition Sciences, University of Queensland, Brisbane, Australia; 4 Postgraduate Program in Epidemiology, Federal University of Pelotas, Brazil; 5 Department of Nutrition, Harvard T.H. Chan School of Public Health, Boston, MA, USA; 6 Department of Epidemiology, Harvard T.H. Chan School of Public Health, Boston, MA, USA; 7 Channing Division of Network Medicine, Brigham and Women’s Hospital and Harvard Medical School, Boston, MA, USA

**Keywords:** epidemiology, non-communicable diseases, physical activity, population attributable fraction, premature mortality

## Abstract

**Background:**

Studies on the impact of counterfactual scenarios of physical activity on premature deaths from non-communicable diseases (NCDs) are sparse in the literature. We estimated preventable premature deaths from NCDs (diabetes, ischemic heart disease, stroke, and breast and colon cancers) in Brazil by increasing population-wide physical activity (i) to theoretical minimum risk exposure levels; (ii) reaching the physical activity recommendation; (iii) reducing insufficient physical activity by 10%; and (iv) eliminating the gender differences in physical activity.

**Methods:**

Preventable fractions were estimated using data from a nationally representative survey, relative risks from a meta-analysis and number of premature deaths (30–69 years) from the Brazilian Mortality Information System.

**Results:**

Physical activity could potentially avoid up to 16 700 premature deaths from NCDs in Brazil, corresponding to 5.75 and 3.23% of premature deaths from major NCDs and of all-causes, respectively. Other scenarios suggested the following impact on premature deaths: reaching physical activity recommendation (5000 or 1.74% of major NCDs); 10% reduction in insufficient physical activity (500 or 0.17% of major NCDs); eliminating gender differences in physical activity (1000 or 0.33% of major NCDs).

**Conclusions:**

Physical activity may play an important role to reduce premature deaths from NCD in Brazil.

## Introduction

‘Death in old age is inevitable, but death before old age is not’.^1^ Under-70 age-standardized mortality rates decreased by 20% in the last decade, but it is still a major concern.^2^ Currently, more than half of global deaths under the age 70 years were from non-communicable diseases (NCDs).^3^ Individuals in low- to middle-income countries face a 1.5 time higher risk of premature mortality from NCD than individuals in high-income countries.^4^ In Brazil, for instance, 40% of all deaths in 2015 occurred between the ages of 30 and 69 years, of which 56% were from major NCDs, namely cardiovascular diseases (CVD), diabetes, cancer and chronic respiratory diseases.^5^

Most of these premature deaths from NCD could be avoided by tackling their major causes.^6^ In 2012, the World Health Organization (WHO) launched the Global Plan for 2025 (hereafter referred to as ‘WHO 25 × 25’) aimed to reduce by 25% premature deaths from NCDs.^3^ To achieve this goal, nine voluntary global targets focusing on some determinants of NCDs (tobacco, alcohol, obesity, physical activity, salt/sodium, hypertension, access to CVD prevention programs and NCD treatments)^3^ were agreed upon.

Physical activity may have an important role for reducing premature deaths from NCDs as it is consistently associated with type 2 diabetes mellitus, ischemic heart disease, stroke, breast cancer and colon cancer.^7–9^ The WHO 25 × 25 target aims a 10% relative reduction in the prevalence of insufficient physical activity (i.e. <600 metabolic equivalent task-minute per week (MET-min/week), usually translated as <150 min per week of moderate to vigorous intensity physical activity).^3^ Reducing the gender differences in the prevalence of insufficient physical activity by increasing physical activity in women has been argued to be an important means toward this WHO target.^10–12^ However, the impact of reaching these physical activity goals on premature deaths from NCDs is unclear.

Herein, we estimated the proportion and number of premature deaths from NCDs that could be avoided in Brazil by increasing physical activity (i) to theoretical minimum risk exposure levels; (ii) to at least 600 MET-min/week; (iii) reducing insufficient physical activity by 10%; and (iv) eliminating the gender differences in physical activity by increasing total physical activity in women to levels observed in men.

## Methods

### Data input

#### Assessment of physical activity

We obtained data on the distribution of physical activity from the National Health Survey (Pesquisa Nacional de Saúde—PNS), 2013.^13,14^ PNS included a nationally representative sample of adult population aged 18 years and over. The PNS sample was randomly selected in three stages: census tracts (primary sampling units), households (second units) and household members 18 years or older (tertiary units). In the final sample, 62 202 adults aged 18 years or over were interviewed (response rate: 86%). In this study, we used information from 55 263 adults aged 18–69 years that responded the physical activity questionnaire. Further information about PNS has been described elsewhere.^13,14^

Physical activity level was assessed using self-reported data of weekly frequency (0–7 days) and duration (hours and minutes) of recreational, occupational, household and commuting activities (walking or cycling to and/or from work; walking or cycling to and/or from other daily activities) in a typical week. Additionally, for individuals engaged in recreational physical activity at least once a week, the most frequent type of recreational activity (e.g. walking, cycling, running, soccer) was asked. The PNS physical activity questionnaire is available in the Supplementary material. We used the compendium of physical activities to assign metabolic equivalent of task (MET) considering the most frequent type of recreational physical activity as well as the average MET for each of the other domains of physical activity (Table S1).^15^ Finally, we estimated total physical activity level by summing up MET-minutes per week (MET-min/week) across domain of physical activity.

### Relative risk estimates

We retrieved relative risks (RRs) and 95% confidence intervals (95% CI) from a recent dose–response meta-analysis of prospective cohort studies^7,16^ for the association between total physical activity (MET-min/week) and risk of diabetes, ischemic heart disease, stroke, breast cancer and colon cancer. The RRs and 95% CI estimates were related to both sexes (except for breast cancer, women only) and all ages combined for the following categories of total physical activity: <600, 600–3999, 4000–7999 and ≥8000 MET-min/week. The reference group (≥8000 MET-min/week) was intended to represent the theoretical minimum risk exposure level, over which there is no additional benefit of physical activity associated with decreased risk of NCDs.^7^ The MET-min/week is equivalent to 5 h/day of moderate intensity physical activities (e.g. brisk walking, walking for transportation).

### Number of NCD premature deaths

We collected the number of premature deaths (age 30–69 years) from diabetes mellitus (ICD E11, E14), ischemic heart disease (ICD I20-I25), stroke (ICD G45 and I64), breast cancer (ICD C50) for women and colon cancer (ICD C18) in 2015 from the Brazilian Mortality Information System.^5^ We additionally obtained number of deaths from all-causes (ICD A00-U99) and major NCDs (diabetes (ICD E10-E14), cardiovascular diseases (ICD I00-I99), cancers (ICD C00-C97) and chronic respiratory diseases (ICD J30-J98)). Major NCDs were selected based on the WHO 25 × 25.^3^

### Data management and analysis

We estimated potential impact fractions (PIF), defined as the expected proportional reduction in deaths that would occur by reducing the exposure in the population, with other conditions remaining the same.^17^ PIF was calculated using the following equation:

$$PIF=\;\frac{\sum_{i=1}^{n}P_{i}\;RR_{i}-\sum_{i=1}^{n}P{'}_{i}\;RR_{i}\;}{\sum_{i=1}^{n}P_{i\;}RR_{i}}$$

where, *P_i_* is the proportion of the population at the level *i* of total physical activity, *P*′*_i_* is the proportion of the population at the level *i* of total physical activity in the counterfactual (alternatively proposed) scenarios, and RR_i_ is the relative risk of each outcome (diabetes, ischemic heart disease, stroke, breast cancer and colon cancer) at the level *i* of total physical activity. Levels *i* of total physical activity were <600, 600–3999, 4000–7999 and ≥8000 MET-min/week.

In this study, we used the following counterfactual scenarios of total physical activity:

- Theoretical minimum risk exposure levels, where everyone reaches at least 8000 MET-min/week. PIF estimates related to this scenario will be hereafter referred as population attributable fraction (PAF), a special case of PIF where the exposure is eliminated.^17,18^
- Physical activity recommendation, where everyone reaches at least 600 MET-min/week.^8^
- 10% relative reduction in the prevalence of insufficient physical activity (<600 MET-min/week).^6^
- Gender equality, where total physical activity level is equal between men and women. In this scenario, we increased physical activity in women to levels observed in men (reference group). Men remained with currently observed physical activity level.

To obtain the number of preventable premature deaths from NCDs, we applied PIF estimates to the number of premature deaths from diabetes, ischemic heart disease, stroke, and breast and colon cancers in 2015.^5^ In addition, we summed up the number of preventable premature deaths from NCDs and divided by total number of premature deaths from major NCD and all-causes.

Finally, we ran a sensitivity analysis considering the current distribution and counterfactual scenarios of recreational and commuting physical activity only. These domains of physical activity have shown the most consistent evidence of preventive effect on NCDs.^9,19,20^ Indeed, interventions and policies aimed to increase population-wide physical activity have focused on recreational and commuting physical activity.^3^

Data analysis was performed in Stata version 15.0. Data input and scripts used in our study are available at https://osf.io/5ut4z/.

## Results

The sample included mostly middle-age adults (mean age 42 years), women (53%), who completed secondary education (41%), white (47%), and were married (44%). Participant characteristics by time spent in total physical activity are shown in Table 1.

**Table 1:** Characteristics of participants by time spent in total physical activity: National Health Survey, 2013, Brazil

Characteristics	Total physical activity (MET-min/week)			
	<600	600–3999	4000–7999	≥8000
	(*n* = 29 482)	(*n* = 22 408)	(*n* = 4051)	(*n* = 3169)
Mean age (years)	41.8	38.7	38.7	36.9
Sex/Gender (%)				
Men	43.5	44.5	60.5	77.8
Women	56.5	55.5	39.5	22.2
Education (%)				
None or incomplete primary	21.0	13.9	17.5	16.8
Complete primary or incomplete secondary	27.7	25.9	33.1	45.6
Complete secondary or incomplete university	38.6	44.2	39.7	33.3
University graduate	12.6	15.9	9.6	4.4
Race/Ethnicity (%)				
White	48.4	47.4	42.2	37.0
Black	8.8	8.9	9.7	14.5
Asian	0.9	0.9	0.9	0.9
Brown	41.7	42.2	46.9	46.9
Native Indian	0.3	0.6	0.3	0.7
Marital status				
Married	46.8	43.1	41.6	35.8
Separated/Divorced	7.2	6.4	6.4	4.9
Widow/widower	5.0	3.3	2.5	3.0
Single	41.1	47.2	49.5	56.4
Domain-specific physical activity (mean MET-min/week)				
Leisure	30	612	1131	937
Occupational	4	224	2667	9182
Commuting	88	536	1059	1049
Household	10	295	747	837

**Fig. 1:**
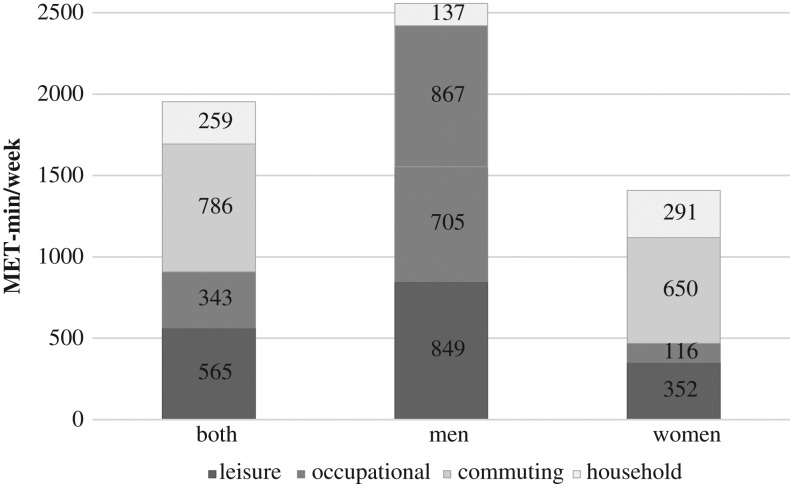
Current physical activity level in metabolic equivalent tasks per min per week (MET-min/week) among Brazilian adults between 18 to 69 years of age, by domain of physical activity and sex.

Only 6.5% of participants reported at least 8000 MET-min/week in total physical activity. This proportion was around 4-fold higher in men (10.6%) than in women (2.7%). Nearly 45% of participants spent <600 MET-min/week in total physical activity, with a higher proportion in women (47.9%) than in men (40.9%) (Table 2). Commuting physical activity (34% men, 46% women) contributed most to total physical activity, followed by recreational (33% men, 25% women), occupational (28% men, 8% women) and household (5% men, 21% women) activities (Fig. 1).

**Table 2:** Current and counterfactual scenarios of physical activity among Brazilian adults between 18 and 69 years of age, by sex

	Physical activity (MET-min/week)			
	<600	600–3999	4000–7999	>8000
Both				
*Current distribution (%)*	44.6	41.3	7.6	6.5
*Counterfactual scenarios (%)*				
Theoretical minimum risk exposure level (≥8000 MET-min/week)	0.0	0.0	0.0	100.0
Physical activity recommendation (≥600 MET-min/week)	0.0	85.9	7.6	6.5
10% reduction in insufficient physical activity	40.2	45.7	7.6	6.5
Gender equality in physical activity	40.9	38.7	9.7	10.6
Men				
*Current distribution (%)*	40.9	38.7	9.7	10.6
*Counterfactual scenarios (%)*				
Theoretical minimum risk exposure level (≥8000 MET-min/week)	0.0	0.0	0.0	100.0
Physical activity recommendation (≥600 MET-min/week)	0.0	79.6	9.7	10.6
10% reduction in insufficient physical activity	36.8	42.8	9.7	10.6
Gender equality in physical activity	40.9	38.7	9.7	10.6
Women				
*Current distribution (%)*	47.9	43.6	5.7	2.7
*Counterfactual scenarios (%)*				
Theoretical minimum risk exposure level (≥8000 MET-min/week)	0.0	0.0	0.0	100.0
Physical activity recommendation (≥600 MET-min/week)	0.0	91.5	5.7	2.7
10% reduction in insufficient physical activity	43.2	48.4	5.7	2.7
Gender equality in physical activity	40.9	38.7	9.7	10.6

The current distribution and counterfactual scenarios of total physical activity by sex are also presented in Table 2. By assuming a 10% relative reduction in insufficient physical activity, the proportion of adults spending <600 MET-min/week would change from 44.6% (40.9% men, 47.9% women) to 40.2% (36.8% men, 43.2% women). If gender-equality scenario were achieved, the prevalence of insufficient physical activity would decrease 15% (absolute difference: 7.0 percentage points) in women and, consequently, 8% (absolute difference: 3.7 percentage points) in both sexes.

In the theoretical minimum risk exposure level scenario, we estimated that around 16 714 premature deaths from NCDs could be avoided by increasing population-wide physical activity. Estimates ranged from 1236 (11.9%) preventable deaths from breast cancer to 4548 (20.2%) preventable deaths from diabetes. Indeed, around 16% or 10 022 premature deaths from CVD (ischemic heart disease and stroke) could be avoided in this scenario. Preventable deaths from all five outcomes (diabetes, ischemic heart disease, stroke, breast cancer and colon cancer) accounted for 5.75% of premature deaths form major NCDs and 3.23% of premature deaths from all-causes (Table 3).

**Table 3:** Preventable premature deaths (from 30 to 69 years) from cancer (breast and colon), diabetes and cardiovascular disease (IHD and stroke) by increasing population-wide physical activity in Brazil

Outcomes	Total premature deaths (*n*)^a^	TMREL (≥8000 MET-min/week)		PA recommendation (≥600 MET-min/week)		10% reduction in insufficient physical activity^b^		Gender equality	
		PAF	Preventable premature deaths (*n*)	PIF	Preventable premature deaths (*n*)	PIF	Preventable premature deaths (*n*)	PIF	Preventable premature deaths (*n*)
Breast cancer									
Both	NA	NA	NA	NA	NA	NA	NA	NA	NA
Men	NA	NA	NA	NA	NA	NA	NA	NA	NA
Women	10 480	11.79	1236	1.65	173	0.16	17	1.18	124
Colon cancer									
Both	5313	17.14	908	4.47	237	0.45	24	1.15	58
Men	2624	16.18	425	4.15	109	0.42	11	0.00	0
Women	2689	17.99	484	4.76	128	0.48	13	2.16	58
Diabetes									
Both	22 618	20.22	4548	7.05	1583	0.70	158	1.47	299
Men	11 748	19.04	2236	6.56	771	0.66	77	0.00	0
Women	10 870	21.26	2311	7.48	813	0.75	81	2.75	299
IHD^c^									
Both	51 566	15.96	8038	4.51	2262	0.45	226	1.17	375
Men	34 514	14.97	5167	4.18	1444	0.42	144	0.00	0
Women	17 048	16.84	2871	4.79	817	0.48	82	2.20	375
Stroke									
Both	11 817	17.00	1984	7.04	819	0.70	82	1.11	102
Men	6917	16.07	1111	6.53	452	0.65	45	0.00	0
Women	4899	17.82	873	7.49	367	0.75	37	2.08	102
Major NCDs									
Both	290 874	5.75	16 714	1.74	5073	0.17	507	0.33	958
Men	163 881	5.45	8939	1.69	2775	0.17	278	0.00	0
Women	126 963	6.12	7775	1.81	2298	0.18	230	0.75	958
All-cause mortality									
Both	517 134	3.23	16 714	0.98	5073	0.10	507	0.19	958
Men	325 583	2.75	8939	0.85	2775	0.09	278	0.00	0
Women	191 477	4.06	7775	1.20	2298	0.12	230	0.50	958

Abbreviation: IHD, Ischemic heart disease. Major NCDs: non-communicable diseases targeted by the World Health Organization (WHO) Global Action Plan for 2025 were diabetes (ICD E10-E14), cardiovascular diseases (ICD I00-I99), cancer (ICD C00-C97) and chronic respiratory diseases (ICD J30-J98); PA, physical activity; TMREL, theoretical minimum risk exposure level; PIF, potential impact fraction.

^a^Nationwide deaths in 2015 among people aged 30–69.

^b^Inactivity defined as ≤600 MET-min/week.

^c^Both sex category does not sum-up sex-specific number of deaths due to missing information about sex (4 deaths for IHD; 1 death for stroke; 74 deaths for all-cause mortality).

Achieving at least the physical activity recommendation (≥600 MET-min/week) could potentially avoid 1.65% (173 deaths) premature deaths from breast cancer and 7.05% (1 583 deaths) from diabetes. Overall, around 5 073 premature deaths could be potentially avoided in this scenario, corresponding to 1.74 and 0.98% of premature deaths from major NCD and all-causes, respectively.

A 10% relative reduction in insufficient physical activity could reduce <1% of all five outcomes, with a total of 507 preventable premature deaths (Table 3).

Increasing physical activity in women to levels observed in men (gender-equality scenario) could potentially avoid, on average, 2% of premature deaths from breast, colon, diabetes, ischemic heart disease and stroke (Table 3).

### Sensitivity analysis

Sensitivity analysis considering only recreational and commuting physical activities suggested slightly higher preventable premature death from NCDs. In these two domains, ~60% of participants spent less than 600 MET-min/week, with a higher proportion in women (62.5%) than in men (56.2%). Only 0.9% of men and 0.2% of women achieved at least 8000 MET-min/week in recreational and commuting physical activities. We estimated that increasing recreational and commuting physical activity could potentially avoid up to 19 259 premature deaths from NCDs in Brazil. Preventable premature deaths represented 6.6% of premature deaths from NCDs and 3.7% of premature deaths from all-causes in Brazil in 2015 (Tables S2–S4).

## Discussion

Physical activity could potentially avoid up to 16 700 premature deaths from NCDs, which correspond to 5.75% of premature deaths from major NCDs and 3.23% of premature deaths from all-causes per year in Brazil. Reaching at least the physical activity recommendation (600 MET-min/week) could potentially avoid 5 000 premature deaths from NCDs per year in Brazil. A 10% relative reduction in insufficient physical activity showed limited impact on premature deaths from NCDs. On the other hand, eliminating gender differences in physical activity showed moderate benefit, estimated to prevent 1000 premature deaths from NCDs.

PAF estimates gained momentum in the physical activity epidemiology literature after the seminal paper published by Lee and colleagues in the first Lancet Physical Activity Series, in 2012.^21^ This study suggested that around 9% or 5.3 million premature deaths worldwide were attributable to physical inactivity.^21^ In the same year, estimates from Global Burden of Disease (GBD) suggested that physical inactivity was responsible for around 6% (3.2 million premature deaths) per year.^22^ In Brazil, PAF estimates were 13.2% by Lee *et al.*^21^ and 2.8% by GBD,^22^ whereas we found that 3.2% of all deaths could be potentially avoided. Differences in PAF estimates could be due to several methodological issues, such as the equations used, source of physical activity data, exposure definition, and RR estimates.^23,24^ In addition, both GBD and our estimates considered cause-specific RR, whereas Lee *et al.* considered all-cause relative risks, to compute the total number of attributable deaths.^23,24^ It is worth noting that all these estimates reflect the theoretical minimum risk exposure level, which despite being informative, is unfeasible in the real world.

Studies on the impact of plausible counterfactual scenarios of physical activity on premature deaths from NCDs are sparse in the literature, despite its clear importance to inform policy makers. We found that achieving the physical activity recommendation may substantially reduce the proportion and number of premature deaths from NCDs, but this recommendation may not be achievable for the entire population.^25,26^ On the other hand, reaching the WHO 25 × 25 target for physical activity (a 10% relative reduction in insufficient physical activity) is likely to have a limited impact on premature deaths from NCDs in Brazil. These results do not necessarily suggest that physical activity should be neither neglected, nor quantitative recommendations ignored. It does, however, reinforce the need for a multisector effort to make physical activity convenient, pleasurable and meaningful,^27^ so more audacious (but feasible) targets could be reached. In addition, the expected net benefit of interventions promoting population-wide physical activity could be substantial given that few negative side effects and non-responders are likely to occur.^28,29^ Finally, these results highlight the importance of concomitantly tackling other determinants of NCDs (e.g. smoking) to reach the 25% reduction in premature deaths from NCDs by 2025.^6^

Gender inequality in physical activity is also an important concern. Activity inequality between countries has been highly driven by the gender gap in physical activity.^12^ Globally, relative and absolute differences in the prevalence of insufficient physical activity are 20% and 7.5 percentage points, respectively, in favor of men.^11^ We found similar figures of gender inequality in the prevalence of insufficient physical activity in Brazil (relative difference: 20%; absolute difference: 7.9 percentage points), and showed that these differences are even more pronounced among those reaching 8000 MET-min/week (relative difference: 390%; absolute difference: 7 percentage points). Yet, most of these differences are driven by occupational physical activity. In Brazil, for instance, 28% of men’s and 8% of women’s total physical activity are occupational activities. Obligatory occupational physical activity has shown less consistent benefit on reduced risk of deaths from NCDs than other domains of physical activity.^9,19,20^ However, heterogeneity in effect sizes between domains of physical activity could be due to residual confounding by socioeconomic status.^30^ We argue that promotion of physical activity should be focused on recreational and commuting physical activity. Currently, recreational physical activity contributes to 33 and 25% of total physical activity in men and women, respectively. Although commuting physical activity contributes to a higher proportion of total physical activity in women (46%), relative to men (34%), there is still absolute differences in the volume of commuting physical activity favoring men (423 MET-min/week in men versus 397 MET-min/week in women). Considering only recreational and commuting physical activity, ~60% of adults spent less than 600 MET-min/week in Brazil. Eliminating gender differences in recreational and commuting physical activity should be considered an important target for increasing physical activity levels and, therefore, preventing premature deaths from NCDs in Brazil.

Our study adds knowledge to estimates of preventable premature deaths from NCDs, especially for policy relevant, plausible counterfactual scenarios of physical activity. We used data from a large nationally representative health survey to estimate the distribution of total physical activity in Brazil and retrieved maximally adjusted RR estimates from a recent dose–response meta-analysis of prospective studies.^7,16^ Physical activity distribution and RR were consistently used in terms of physical activity construct (total physical activity, in MET-min/week) and cut-offs (<600, 600–3999, 4000–7999 and ≥8000 MET-min/week). Nonetheless, there are several data limitations. The current physical activity level was assessed using questionnaire and did not include information about the intensity of physical activities. However, we used the 2011 compendium of physical activities to assign average METs to self-reported recreational physical activities as well as other domain of physical activity. RR estimates from the dose–response meta-analysis^7,16^ were derived mainly from USA and European cohort studies and whether they are applicable to the Brazilian population is unknown. These RR estimates were not available by type (e.g. aerobic and resistance), domain (recreational, occupational, commuting and household) and intensity (e.g. moderate and vigorous) of activities. Despite using multiple RR categories of total physical activity, these estimates did not capture potential benefits of shifting from inactivity to low physical activity levels (e.g. 300 MET-min/week) on premature deaths from NCDs. Some RR included in the dose–response meta-analysis were adjusted by potential mediators (e.g. body mass index, systolic blood pressure) through which physical activity may reduce the risk of NCDs. These limitations may have underestimated the impact of physical activity on premature deaths from NCDs. In addition, physical activity was self-reported rather than objectively measured, thus biases arising from measurement error is likely to have occurred. Finally, we restricted our analysis to prevention of premature deaths from NCDs targeted in the WHO 25 × 25, but physical activity may confer further benefits on other NCDs (e.g. dementia)^31^ as well as overall population health.^8^

Physical activity may play an important role to reduce premature deaths from NCDs in Brazil. The WHO target for physical activity (10% relative reduction in insufficient physical activity) might be a feasible goal but suggested a limited impact on prevention of premature deaths from NCDs. On the other hand, interventions and policies aiming at tackling structural determinants of gender inequality in physical activity might confer a moderate impact on premature deaths from NCD in Brazil.

## Supplementary Material

fdy183_Supplemetary_material
