# Supplementary material for: Physical activity and preventable premature deaths from non-communicable diseases in Brazil
Source: J Public Health (Oxf). 2018 Oct 20;41(3):e253–60. doi: 10.1093/pubmed/fdy183 (PMC6785690; doi:10.1093/pubmed/fdy183)
Supplement: fdy183_Supplemetary_material [file fdy183_supplemetary_material.docx]

**SUPPLEMENTARY MATERIAL**

**National Health Survey 2013, Physical activity Questionnaire**

**Recreational activities**

***P34. In the last three months, have you practiced any kind of physical exercise or sport? (do not consider physical therapy)***

Response: Yes or No

***P35. How many days a week do you usually exercise or sport?***

Response: (0 to 7) days a week

***P36. What physical exercise or sport do you most often practice?***

Response: Walking; Walking treadmill; Running/cooper; Running treadmill; Weight training; Aerobic classes; Water aerobics; Gym/Pilates/Yoga; Swimming; Martial arts; Cycling; Soccer; Basketball; Volleyball; Tennis; Dance; Other (specify which).

***P37. In general, on the day you practice exercise or sport, how long does this activity last?***

Response: _ _ Hours _ _ Minutes

**Occupational activities**

***P38. At your job, do you walk a lot?***

Response: Yes or No

***P39. At your job, do you do heavy cleaning, carry weight, or do other heavy activity that requires intense physical exertion?***

Response: Yes or No

***P39a. In a typical week, how many days do you do these activities at work?***

Response: (0 to 7) days a week

***P39b. How much time do you spend doing physical activity on a normal work day?***

Response: _ _ Hours _ _ Minutes

**Commuting activities**

***P40. To go to or return from work, do you do any walking or cycling? (Read the answer options)***

Response: Yes, the whole way; Yes, part of the way; No

***P41. How much time do you spend per day to go this route on foot or by bicycle, considering the trip to and from work?***

Response: _ _ Hours _ _ Minutes

***P42. In your usual activities (such as going to a course, school or club or taking someone to a course, school or club), how many days per week do you do any activity that involves walking or cycling?***

Response: (0 to 7) days a week

***P43. No dia em que o(a) sr(a) faz esta atividade, quanto tempo o(a) sr(a) gasta no deslocamento a pé ou de bicicleta, considerando a ida e a volta?***

Response: _ _ Hours _ _ Minutes

**Household activities**

***P44. In your household activities, do you do heavy cleaning, carry weight, or do other heavy activity that requires intense physical exertion?***

Response: Yes or No

***P44a. In a typical week, in your household activities, on how many days do you do heavy cleaning or do activities that require intense physical exertion?***

Response: (0 to 7) days a week

***P44b. How much time do you spend per day doing these heavy domestic activities?***

Response: _ _ Hours _ _ Minutes

**Table S1**: Metabolic equivalent of tasks (MET) assigned for each physical activity domain.

| **Physical activity domain** | **2011 Compendium Code** | **MET** | **Comment** |
| --- | --- | --- | --- |
| **Recreational** |  |  |  |
| Walking | 17190 | 3.5 |  |
| Walking treadmill | 17190 | 3.5 |  |
| Running/cooper | 12030 | 8.3 |  |
| Running treadmill | 12030 | 8.3 |  |
| Weight training | 02054 | 3.5 |  |
| Aerobic classes | 02062 | 7.8 |  |
| Water aerobics | 18355 | 5.5 |  |
| Gym/Pilates/Yoga | 02061/02105/02101/02160/02170 | 3.5 | mean value |
| Swimming | 18240 | 5.8 |  |
| Martial arts | 15425/15430 | 7.8 | mean value |
| Cycling | 02017 | 4.8 |  |
| Soccer | 15610 | 7 |  |
| Basketball | 15055 | 6.5 |  |
| Volleyball | 15720 | 3 |  |
| Tennis | 15675 | 7.3 |  |
| Dance | 03040 | 3 |  |
| Other |  | 3.5 | mean value for MVPA |
| **Occupation** |  |  |  |
| Active commute during work | 11792 | 3.5 |  |
| Lift weight during work | 11630 | 4.5 |  |
| **Active Commuting** |  |  |  |
| Active commute to work | 16060/01010 | 3.5 | mean value |
| **Household** |  |  |  |
| Cleaning house | 05021 | 3.5 |  |

Source: Ainsworth BE, Haskell WL, Herrmann SD et al. The compendium of physical activities tracking guide. Healthy Lifestyles Research Center, College of Nursing & Health Innovation, Arizona State University. Retrieved May 3rd, 2017, from the World Wide Web. <https://sites.google.com/site/compendiumofphysicalactivities/>; Abbreviation: MVPA: moderate to vigorous physical activities

**Table S2**: Characteristics of participants by time spent in recreational and commuting physical activities: National Health Survey, 2013. Brazil.

|  | **Recreational and Commuting physical activity**  **(MET-min/week)** | | | |
| --- | --- | --- | --- | --- |
| **Characteristics** | **<600** | **600 to 3,999** | **4,000 to 7,999** | **≥8,000** |
|  | (n=37,480) | (n=20,070) | (n=1,335) | (n=225) |
| **Mean age (years)** | 41.4 | 38.2 | 35.7 | 32.1 |
| **Sex/Gender (%)** |  |  |  |  |
| Men | 44.8 | 50.6 | 55.9 | 81.3 |
| Women | 55.2 | 49.4 | 44.1 | 18.7 |
| **Education (%)** |  |  |  |  |
| None or incomplete primary | 20.2 | 13.7 | 12.2 | 9.6 |
| Complete primary or incomplete secondary | 29.8 | 26.7 | 25.5 | 35.4 |
| Complete secondary or incomplete university | 38.6 | 43.5 | 46.7 | 42.9 |
| University Graduate | 11.3 | 16.1 | 15.5 | 12.1 |
| **Race/Ethnicity (%)** |  |  |  |  |
| White | 47.4 | 46.5 | 36.3 | 36.2 |
| Black | 9.1 | 9.4 | 10.8 | 11.7 |
| Asian | 0.9 | 0.9 | 1.9 | 0.0 |
| Brown | 42.3 | 42.6 | 50.3 | 51.9 |
| Native Indian | 0.4 | 0.6 | 0.6 | 0.2 |
| **Marital Status** |  |  |  |  |
| Married | 47.1 | 40.5 | 32.0 | 27.8 |
| Separated/Divorced | 6.9 | 6.3 | 6.6 | 3.1 |
| Widow/widower | 4.5 | 3.2 | 2.1 | 2.4 |
| Single | 41.5 | 50.0 | 59.2 | 66.6 |
| **Domain-specific physical activity**  **(mean MET-min/week)** | |  |  |  |
| Recreational | 31 | 760 | 2926 | 7000 |
| Commuting | 100 | 713 | 2415 | 4195 |

| **Table S3**: Current and counterfactual scenarios of recreational and commuting physical activity among Brazilian adults between 18 to 69 years of age, by sex. | | | | |
| --- | --- | --- | --- | --- |
|  | Recreational and Commuting physical activity  (MET-min/week) | | | |
|  | <600 | 600-3999 | 4000-7999 | >8000 |
| **Both** |  |  |  |  |
| *Current distribution (%)* | 59.5 | 37.4 | 2.6 | 0.5 |
| *Counterfactual Scenarios (%)* |  |  |  |  |
| Theoretical minimum risk exposure level (≥8000 MET-min/week) | 0.0 | 0.0 | 0.0 | 100.0 |
| Physical activity recommendation (≥600 MET-min/week) | 0.0 | 96.9 | 2.6 | 0.5 |
| 10% reduction in insufficient physical activity | 53.6 | 43.4 | 2.6 | 0.5 |
| Gender equality in physical activity | 56.2 | 39.9 | 3.0 | 0.9 |
|  |  |  |  |  |
| **Men** |  |  |  |  |
| *Current distribution (%)* | 56.2 | 39.9 | 3.0 | 0.9 |
| *Counterfactual Scenarios (%)* |  |  |  |  |
| Theoretical minimum risk exposure level (≥8000 MET-min/week) | 0.0 | 0.0 | 0.0 | 100.0 |
| Physical activity recommendation (≥600 MET-min/week) | 0.0 | 96.1 | 3.0 | 0.9 |
| 10% reduction in insufficient physical activity | 50.6 | 45.5 | 3.0 | 0.9 |
| Gender equality in physical activity | 56.2 | 39.9 | 3.0 | 0.9 |
|  |  |  |  |  |
| **Women** |  |  |  |  |
| *Current distribution (%)* | 62.5 | 35.2 | 2.1 | 0.2 |
| *Counterfactual Scenarios (%)* |  |  |  |  |
| Theoretical minimum risk exposure level (≥8000 MET-min/week) | 0.0 | 0.0 | 0.0 | 100.0 |
| Physical activity recommendation (≥600 MET-min/week) | 0.0 | 97.7 | 2.1 | 0.2 |
| 10% reduction in insufficient physical activity | 56.3 | 41.4 | 2.1 | 0.2 |
| Gender equality in physical activity | 56.2 | 39.9 | 3.0 | 0.9 |

**Table S4**: Proportion and number of cancer (breast and colon), diabetes, and cardiovascular disease (IHD and stroke) premature deaths (from 30 to 70 years) that could be avoided in Brazil by increasing population-wide recreational and commuting physical activity.

| **Outcomes** | **Total**  **Premature deaths (n)^*^** |  | **TMREL**  **(≥ 8,000 MET-min/week)** | |  | **PA recommendation**  **(≥ 600 MET-min/week)** | |  | **10% reduction in insufficient**  **physical activity**** | |  | **Gender equality in physical activity** | |
| --- | --- | --- | --- | --- | --- | --- | --- | --- | --- | --- | --- | --- | --- |
|  |  |  | **PAF** | **Preventable**  **Premature Deaths (n)** |  | **PIF** | **Preventable**  **Premature Deaths (n)** |  | **PIF** | **Preventable**  **Premature Deaths (n)** |  | **PIF** | **Preventable**  **Premature Deaths (n)** |
| **Breast cancer** |  |  |  |  |  |  |  |  |  |  |  |  |  |
| Both | NA |  | NA | NA |  | NA | NA |  | NA | NA |  | NA | NA |
| Men | NA |  | NA | NA |  | NA | NA |  | NA | NA |  | NA | NA |
| Women | 10,480 |  | 12.55 | 1,315 |  | 2.13 | 223 |  | 0.21 | 22 |  | 0.31 | 33 |
| **Colon cancer** |  |  |  |  |  |  |  |  |  |  |  |  |  |
| Both | 5,313 |  | 19.38 | 1,029 |  | 5.81 | 308 |  | 0.58 | 31 |  | 0.41 | 21 |
| Men | 2,624 |  | 19.05 | 500 |  | 5.50 | 144 |  | 0.55 | 14 |  | 0.00 | 0 |
| Women | 2,689 |  | 19.68 | 529 |  | 6.07 | 163 |  | 0.61 | 16 |  | 0.78 | 21 |
| **Diabetes** |  |  |  |  |  |  |  |  |  |  |  |  |  |
| Both | 22,618 |  | 23.19 | 5,236 |  | 9.05 | 2,038 |  | 0.91 | 204 |  | 0.61 | 126 |
| Men | 11,748 |  | 22.72 | 2,669 |  | 8.60 | 1,010 |  | 0.86 | 101 |  | 0.00 | 0 |
| Women | 10,870 |  | 23.62 | 2,567 |  | 9.45 | 1,028 |  | 0.95 | 103 |  | 1.16 | 126 |
| **IHD^#^** |  |  |  |  |  |  |  |  |  |  |  |  |  |
| Both | 51,566 |  | 18.32 | 9,380 |  | 5.84 | 2,955 |  | 0.58 | 296 |  | 0.43 | 137 |
| Men | 34,514 |  | 17.97 | 6,203 |  | 5.54 | 1,913 |  | 0.55 | 191 |  | 0.00 | 0 |
| Women | 17,048 |  | 18.63 | 3,177 |  | 6.12 | 1,043 |  | 0.61 | 104 |  | 0.81 | 137 |
| **Stroke** |  |  |  |  |  |  |  |  |  |  |  |  |  |
| Both | 11,817 |  | 19.55 | 2,298 |  | 9.10 | 1,064 |  | 0.91 | 106 |  | 0.56 | 52 |
| Men | 6,917 |  | 19.09 | 1,321 |  | 8.64 | 598 |  | 0.86 | 60 |  | 0.00 | 0 |
| Women | 4,899 |  | 19.95 | 978 |  | 9.51 | 466 |  | 0.95 | 47 |  | 1.06 | 52 |
| **Major NCDs** |  |  |  |  |  |  |  |  |  |  |  |  |  |
| Both | 290,874 |  | 6.62 | 19,259 |  | 2.26 | 6,588 |  | 0.23 | 659 |  | 0.13 | 369 |
| Men | 163,881 |  | 6.53 | 10,693 |  | 2.24 | 3,665 |  | 0.22 | 366 |  | 0.00 | 0 |
| Women | 126,963 |  | 6.75 | 8,566 |  | 2.30 | 2,923 |  | 0.23 | 292 |  | 0.29 | 369 |
| **All-cause mortality** |  |  |  |  |  |  |  |  |  |  |  |  |  |
| Both | 517,134 |  | 3.72 | 19,259 |  | 1.27 | 6,588 |  | 0.13 | 659 |  | 0.07 | 659 |
| Men | 325,583 |  | 3.28 | 10,693 |  | 1.13 | 3,665 |  | 0.11 | 366 |  | 0.00 | 366 |
| Women | 191,477 |  | 4.47 | 8,566 |  | 1.53 | 2,923 |  | 0.15 | 292 |  | 0.19 | 292 |
| * Nationwide deaths in 2015 among people aged 30 to 69. **Insufficient physical activity defined as ≤600 MET-min/week;  Abbreviation: IHD: Ischemic heart disease. Major NCDs: Non-communicable diseases targeted by the World Health Organization (WHO) Global Action Plan for 2025 were diabetes (ICD E10-E14), cardiovascular diseases (ICD I00-I99), cancer (ICD C00-C97), and chronic respiratory diseases (ICD J30-J98); PA: physical activity; TMREL: theoretical minimum risk exposure level; PAF: Population attributable fraction; PIF: Potential impact fraction.  **^#^**Both sex category does not sum-up sex-specific number of deaths due to missing information about sex (4 deaths for IHD; 1 death for Stroke; 74 deaths for all-cause mortality). | | | | | | | | | | | | | |
